# Identification of Endogenous Substances Involved in Sclareol-Induced Chlorophyll Reductions in *Arabidopsis*

**DOI:** 10.3390/plants15152301

**Published:** 2026-07-27

**Authors:** Asma Ben Hmidene, Shigemi Seo

**Affiliations:** Department of Plant-Microbe Interactions, Institute of Agrobiological Sciences, National Agriculture and Food Research Organization, 1-2 Owashi, Tsukuba 305-8634, Ibaraki, Japan

**Keywords:** sclareol, *Arabidopsis*, chlorophyll reduction, α-pinene, oleic acid, triolein, pipecolic acid

## Abstract

Sclareol, a natural diterpene, exhibits diverse physiological activities in plants, microorganisms, and animals. Exogenous application of sclareol to *Arabidopsis thaliana* leaves induces chlorosis-like symptoms accompanied by a reduction in chlorophyll content. In our previous study, a bioassay-guided fractionation approach was employed to isolate endogenous compounds responsible for this decrease, leading to the identification of campesterol and stigmasterol as active phytosterols. Notably, this approach also indicated the presence of additional active substances in fractions lacking these phytosterols. In the present study, we identified α-pinene, oleic acid, triolein, and pipecolic acid as additional compounds capable of reducing chlorophyll content. Exogenous application of each compound to *Arabidopsis* leaves resulted in a dose-dependent decline in chlorophyll levels. Furthermore, sclareol treatment increased the endogenous accumulation of these metabolites, along with the expression of genes involved in their biosynthesis. Because phytosterols, terpenoids, lipids, and pipecolic acid have been implicated in plant growth and development, stress responses, and disease resistance, the metabolites identified in this study are likely to contribute not only to sclareol-induced chlorophyll reduction but also to other physiological responses elicited by sclareol. Collectively, these findings suggest that sclareol triggers coordinated metabolic reprogramming in *Arabidopsis*, leading to the accumulation of multiple bioactive metabolites that mediate diverse physiological processes.

## 1. Introduction

**Figure 1:**
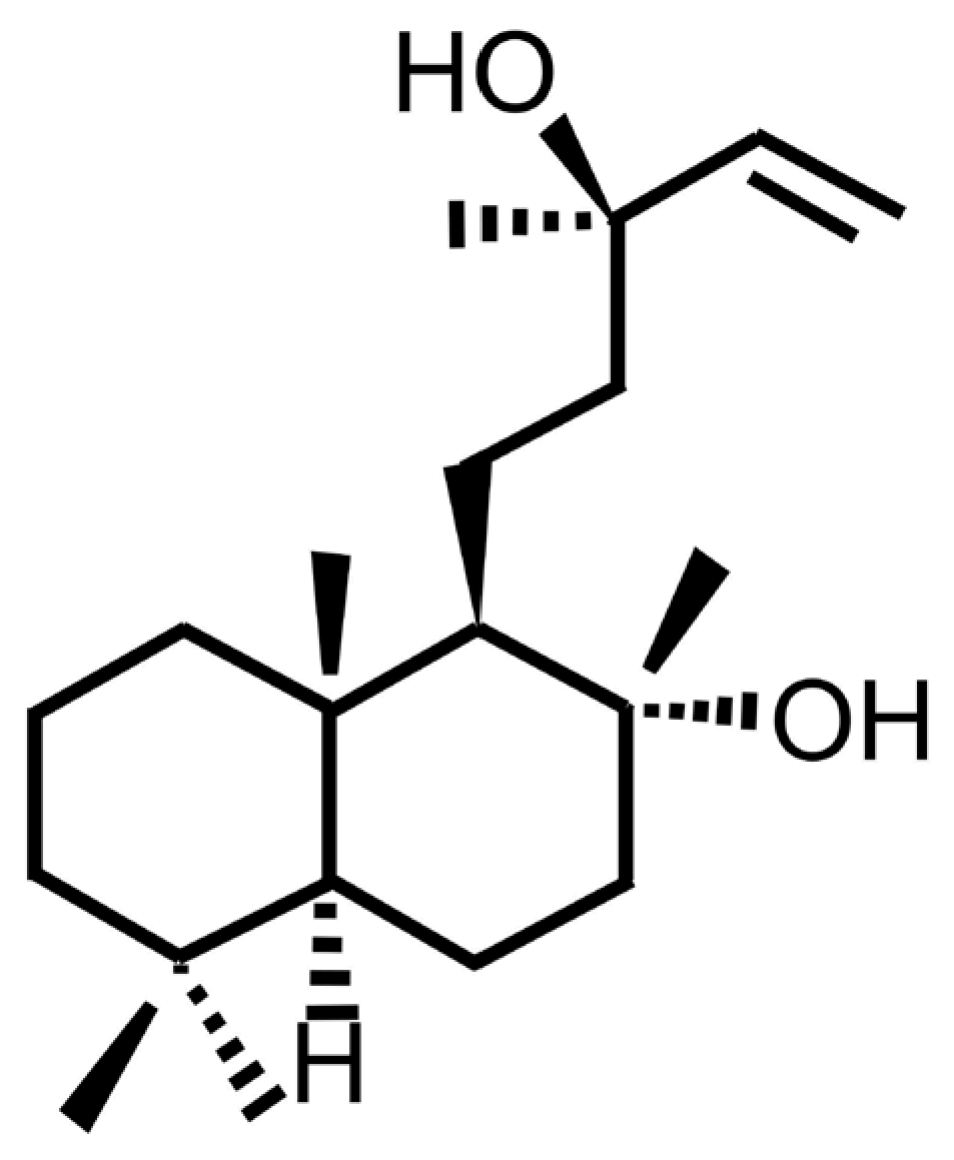
Chemical structure of sclareol.

Diterpenes constitute a major class of terpenoids composed of four isoprene units and play diverse roles in higher plants, functioning as phytohormones, growth regulators, antioxidants, and defense-related molecules. Among them, sclareol (Figure 1), an aromatic labdane-type diterpene, has been identified in a limited number of plant species, including clary sage (*Salvia sclarea*) and tobacco (*Nicotiana tabacum*) [1,2,3,4,5,6,7,8]. Owing to its characteristic fragrance, sclareol is widely utilized in the flavor and fragrance industry. In addition to its industrial relevance, sclareol exhibits a broad spectrum of biological activities across microorganisms, animals, and plants [9].

Previous studies have demonstrated that sclareol possesses antimicrobial activity against plant pathogenic fungi and bacteria [1,2,5,10]. In animal systems, pharmacological treatment with sclareol induces multiple cellular responses, including apoptosis, inhibition of inflammation, and suppression of tumor growth in both mouse and human cells [9,11,12,13]. In plants, exogenous application of sclareol has been reported to inhibit coleoptile and root growth, promote anthocyanin accumulation, and induce the expression of defense-related genes in several species, including *Arabidopsis thaliana*, tobacco (*N. tabacum*), and wheat (*Triticum aestivum*) [8,14,15,16,17]. Furthermore, sclareol treatment enhances resistance to plant diseases, such as those caused by bacterial pathogens and nematodes, in *Arabidopsis*, tobacco, and tomato (*Solanum lycopersicum*), despite lacking direct antibacterial or antinematicidal activity [8,18]. Genetic and pharmacological analyses have indicated that this induced resistance is partially mediated through ethylene-dependent signaling pathways [8,18].

Notably, exogenous application of sclareol to *Arabidopsis*, a species that does not endogenously produce this compound, results in a reduction in chlorophyll content [18], suggesting that sclareol triggers the accumulation of endogenous factors responsible for chlorophyll degradation. In our previous work, we detected chlorophyll-reducing activity in acetone extracts of sclareol-treated *Arabidopsis* tissues and, through bioassay-guided fractionation, identified the phytosterols campesterol and stigmasterol as active compounds [19]. However, additional fractions lacking these phytosterols also exhibited chlorophyll-reducing activity, indicating the presence of other unidentified metabolites.

In the present study, we report the isolation and identification of additional metabolites that contribute to the reduction in chlorophyll content in *Arabidopsis*.

## 2. Results

### 2.1. Purification and Isolation of Substances That Reduce the Content of Chlorophyll

In our previous studies, root application of 100 μM sclareol for 48 h was sufficient to induce both chlorophyll reduction and disease resistance in *Arabidopsis* plants [18,19]. Because treatment for 72 h or longer frequently impaired total RNA extraction from treated plants, a 48-h treatment period was used for subsequent analyses. Hexane-, ethyl acetate-, and water-soluble fractions were prepared from acetone extracts of sclareol-treated plants and evaluated for chlorophyll content-reducing activity. Although all three fractions showed activity, the activity in the water-soluble fraction was lost after further fractionation. Therefore, subsequent purification and identification of active compounds were focused on the hexane- and ethyl acetate-soluble fractions.

The hexane-soluble fraction was subjected to silica gel column chromatography using a hexane–ethyl acetate solvent system. Chlorophyll content-reducing activity was detected in fractions eluted with 30–50% ethyl acetate from sclareol-treated plants, whereas no such activity was observed in fractions derived from control plants (0.1% methanol) (Supplemental Figure S1A,B). These findings are consistent with our previous report [19].

**Figure 2:**
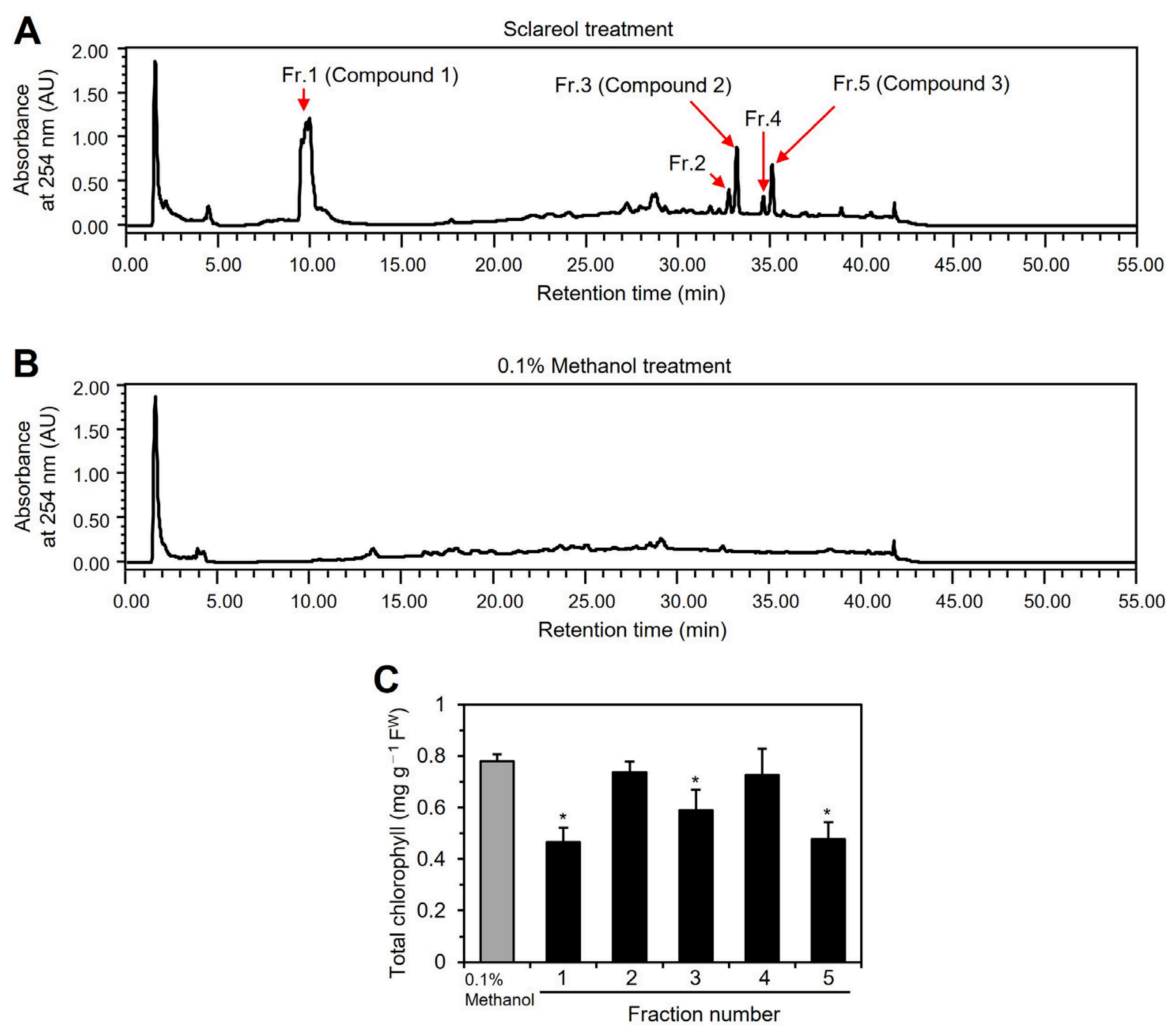
Separation of chlorophyll content-reducing substances in the hexane-soluble fraction by HPLC. (**A**,**B**) Representative HPLC chromatograms of fractions derived from *Arabidopsis* plants treated with sclareol (**A**) or 0.1% (*v*/*v*) methanol ((**B**), control). (**C**) Effects of HPLC fractions (Fr. 1–5) on chlorophyll content. Total chlorophyll represents the sum of chlorophyll *a* and *b*. Data are presented as means ± standard deviation (SD) of three biological replicates. Asterisks indicate significant differences compared with the control (0.1% methanol; Dunnett’s test, *p* < 0.05).

Because the 30% ethyl acetate fraction contained the previously identified phytosterols campesterol and stigmasterol, further purification focused on the 40% and 50% ethyl acetate fractions. These fractions were combined and subjected to reversed-phase high-performance liquid chromatography (HPLC). This analysis revealed several peaks specific to sclareol-treated samples (Fractions 1–5 in Figure 2A compared with Figure 2B). Among these, fractions 1, 3, and 5 exhibited chlorophyll content-reducing activity (Figure 2C).

**Figure 3:**
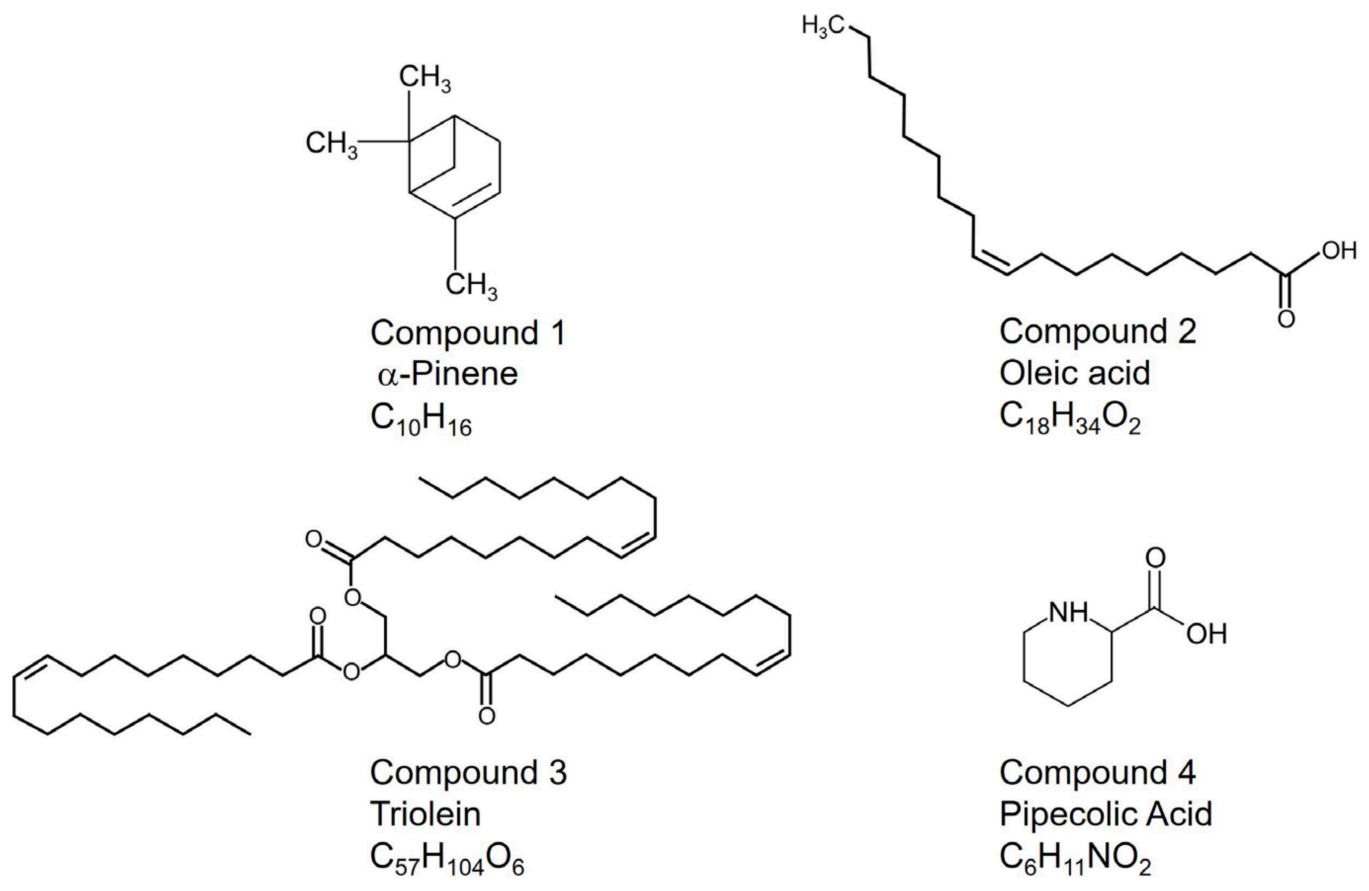
Chemical structures of chlorophyll content-reducing substances. Structures of α-pinene (compound 1), oleic acid (compound 2), triolein (compound 3), and pipecolic acid (compound 4).

These three active fractions were collected and subjected to structural characterization. Based on ^1^H and ^13^C nuclear magnetic resonance (NMR) spectroscopic analyses, the compounds present in fractions 1, 3, and 5 were identified as the monoterpene α-pinene (compound 1 in Figure 3), the unsaturated fatty acid oleic acid (compound 2), and the triacylglycerol (TAG) triolein (compound 3), respectively.

The ethyl acetate-soluble fraction was further separated by silica gel column chromatography using a chloroform–methanol solvent system. Chlorophyll content-reducing activity was detected exclusively in the 100% methanol fraction derived from sclareol-treated plants, whereas no activity was observed in the corresponding fraction from control plants (Supplemental Figure S1A,C).

**Figure 4:**
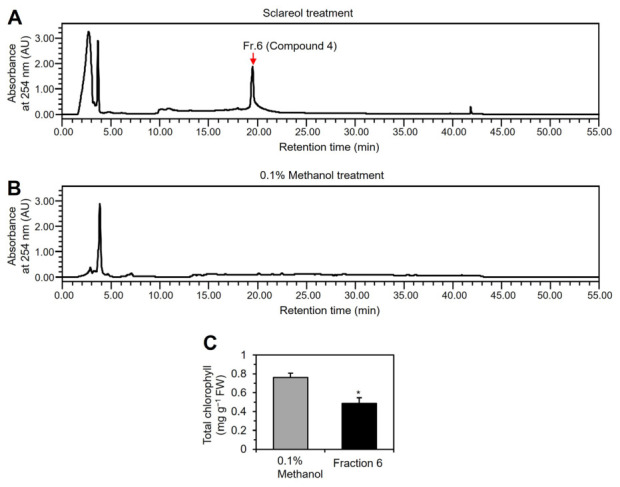
Separation of chlorophyll content-reducing substances in the ethyl acetate-soluble fraction by HPLC. (**A**,**B**) Representative HPLC chromatograms of fractions derived from *Arabidopsis* plants treated with sclareol (**A**) or 0.1% (*v*/*v*) methanol ((**B**), control). (**C**) Effect of the HPLC fraction (Fr. 6) on chlorophyll content. Total chlorophyll represents the sum of chlorophyll *a* and *b*. Data are presented as means ± standard deviation (SD) of three biological replicates. Asterisks indicate significant differences compared with the control (0.1% methanol; Student’s *t*-test, *p* < 0.05).

The active fraction was subsequently subjected to reversed-phase HPLC. A peak specific to sclareol-treated samples (Fraction 6 in Figure 4A compared with Figure 4B) exhibited chlorophyll-reducing activity (Figure 4C) and was further purified. Structural characterization based on ^1^H and ^13^C NMR analyses identified the compound in Fraction 6 as pipecolic acid, a non-proteinogenic amino acid (compound 4 in Figure 3).

**Figure 5:**
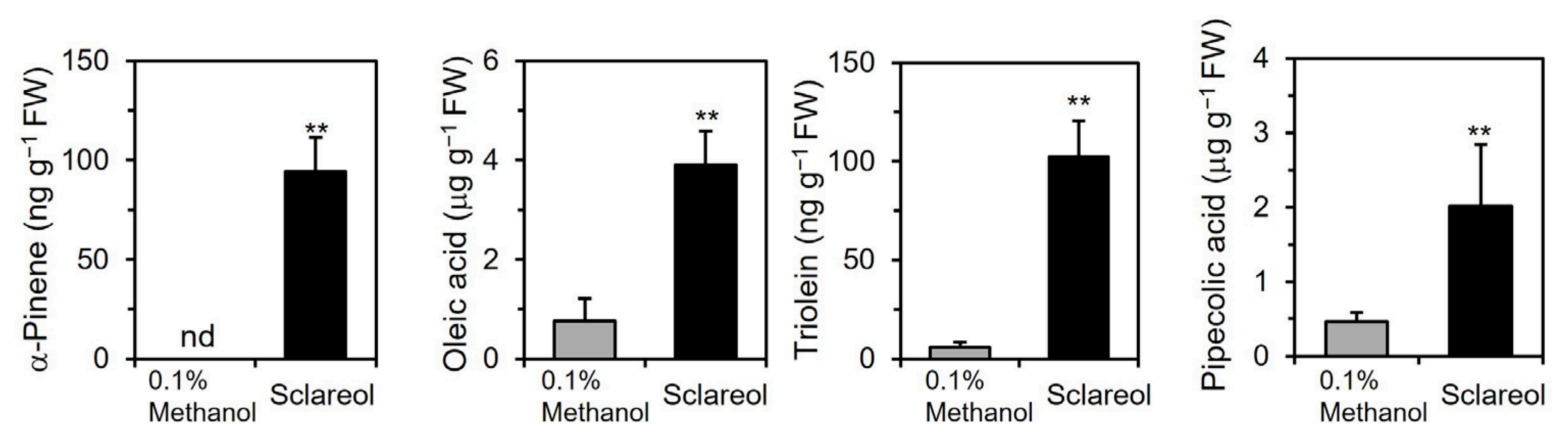
Effects of exogenous sclareol on the accumulation of chlorophyll content-reducing substances in *Arabidopsis* plants. Endogenous levels of α-pinene, oleic acid, triolein, and pipecolic acid in aerial tissues of plants treated with 100 µM sclareol or 0.1% (*v*/*v*) methanol (control) for 48 h. Data are presented as means ± standard deviation (SD) of three biological replicates. Asterisks indicate significant differences compared with the control (0.1% methanol; Student’s *t*-test, *p* < 0.01). The experiment was repeated three times with similar results.

Quantitative analyses revealed that the endogenous levels of oleic acid, triolein, and pipecolic acid were significantly elevated in sclareol-treated leaves compared with control leaves (Figure 5). Although α-pinene was not detected in control leaves under the present experimental conditions, it was clearly detected in sclareol-treated leaves.

### 2.2. Effects of α-Pinene, Oleic Acid, Triolein, and Pipecolic Acid on Chlorophyll Reductions

**Figure 6:**
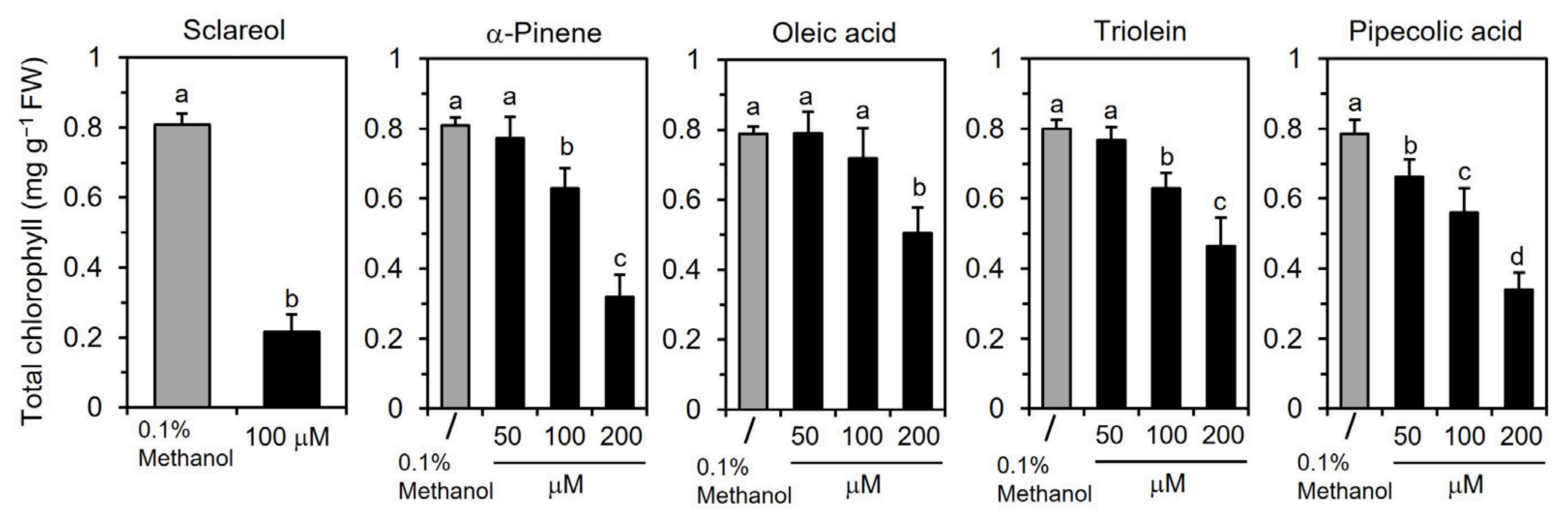
Effects of α-pinene, oleic acid, triolein, and pipecolic acid on chlorophyll content in *Arabidopsis* plants. Chlorophyll levels in aerial tissues of plants treated with the indicated compounds for 48 h. Total chlorophyll represents the sum of chlorophyll *a* and *b*. The control treatment consisted of 0.1% (*v*/*v*) methanol. Data are presented as means ± standard deviation (SD) of three biological replicates. Different letters indicate statistically significant differences among treatments (Tukey’s test, *p* < 0.05). The experiment was repeated three times with similar results.

To evaluate the dose dependency of α-pinene, oleic acid, triolein, and pipecolic acid on chlorophyll reduction, *Arabidopsis* plants were treated via root application with solutions containing varying concentrations of each compound, and chlorophyll content in the leaves was subsequently measured. The effects of α-pinene and triolein became apparent at concentrations of 100 µM or higher (Figure 6). Oleic acid reduced chlorophyll content at 200 µM, whereas pipecolic acid exhibited chlorophyll-reducing activity at concentrations of 50 µM or higher.

### 2.3. Effects of Sclareol on Expression of Biosynthetic Genes of Chlorophyll-Content-Reducing Substances

**Figure 7:**
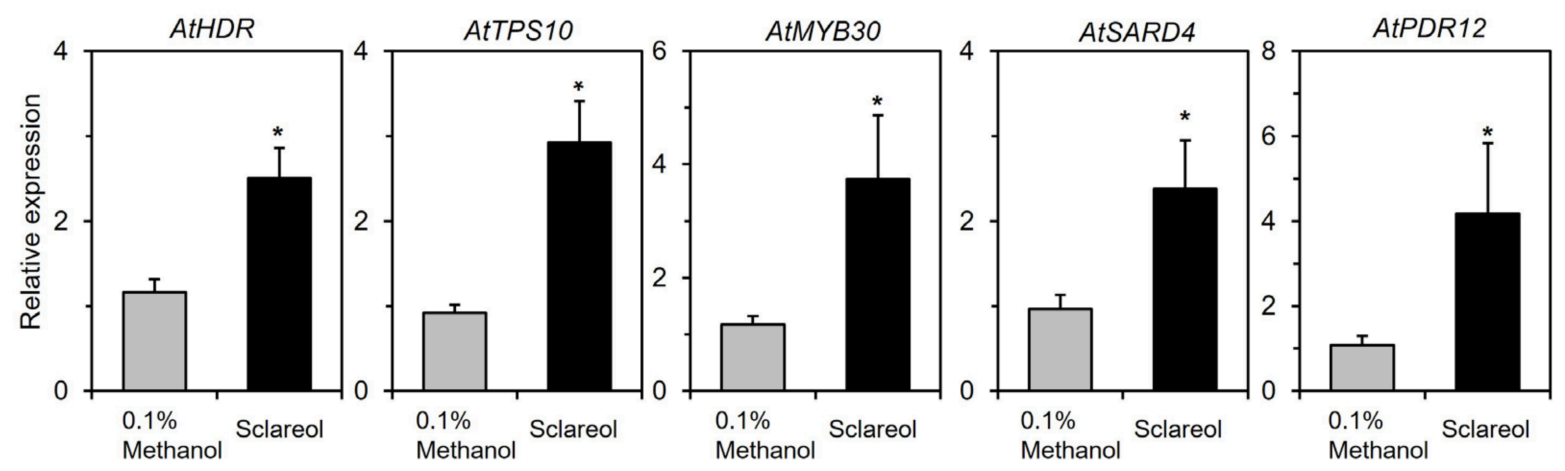
Effects of exogenous sclareol on the expression of secondary metabolite biosynthetic genes in Arabidopsis plants. Relative transcript levels of the indicated genes were analyzed by real-time quantitative PCR (qPCR) using aerial tissues of plants treated with 100 µM sclareol or 0.1% (*v*/*v*) methanol (control) for 48 h. Gene expression levels were normalized to AtACTIN2. Data are presented as means ± standard deviation (SD) of three biological replicates. Asterisks indicate significant differences compared with the control (0.1% methanol; Student’s *t*-test, *p* < 0.05). The experiment was repeated three times with similar results.

To examine whether the sclareol-induced accumulation of α-pinene, oleic acid, triolein, and pipecolic acid is associated with the activation of their biosynthetic pathways, the expression dynamics of related genes were analyzed in sclareol-treated *Arabidopsis* leaves. The genes examined included *AtHDR*, encoding 4-hydroxy-3-methylbut-2-enyl diphosphate reductase, a key enzyme in the methylerythritol 4-phosphate (MEP) pathway for terpenoid biosynthesis [20,21,22]; *AtTPS10*, a member of the TPS-b subfamily involved in monoterpene biosynthesis, including α-pinene [23,24,25,26]; *AtMYB30*, a transcription factor regulating fatty acid and lipid biosynthesis [27]; and *AtSARD4*, which is required for pipecolic acid biosynthesis [28]. Transcript accumulation of *AtHDR*, *AtTPS10*, *AtMYB30*, and *AtSARD4* was markedly enhanced in response to sclareol treatment (Figure 7). In addition, increased expression of *AtPDR12*, a known sclareol-responsive gene [8,15], was confirmed in sclareol-treated leaves, validating the effectiveness of the experimental system.

## 3. Discussion

Terpenoids constitute a large and diverse class of natural products derived from isoprene units and include compounds such as monoterpenes, sesquiterpenes, diterpenes, triterpenes, carotenoids, and phytosterols. In plants, all terpenoids originate from isopentenyl diphosphate (IPP) and dimethylallyl diphosphate (DMAPP), which are synthesized via the cytosolic mevalonic acid (MVA) pathway and the plastidial methylerythritol 4-phosphate (MEP) pathway. Generally, sesquiterpenes, triterpenes, and phytosterols are produced via the MVA pathway, whereas monoterpenes, diterpenes, and carotenoids are generated through the MEP pathway.

In the present study, exogenous application of sclareol enhanced the accumulation of α-pinene, a monoterpene, and increased the expression of its biosynthetic gene, *AtTPS10*. Together with the induction of *AtHDR*, which encodes a key enzyme in the methylerythritol 4-phosphate (MEP) pathway, these findings suggest that sclareol promotes monoterpene biosynthesis through transcriptional activation of the MEP pathway. In our previous study, campesterol and stigmasterol were identified as endogenous metabolites contributing to the reduction in chlorophyll content induced by sclareol in *Arabidopsis* [19]. Their accumulation was accompanied by the upregulation of biosynthetic genes, including those encoding 3-hydroxy-3-methylglutaryl-CoA reductase (HMGR), sterol 4α-methyl oxidase 1 (SMO1-2), and sterol C-24 reductase (DWF1). Collectively, these findings indicate that sclareol promotes transcriptional activation of both the MVA and MEP pathways, leading to increased accumulation of α-pinene and the phytosterols campesterol and stigmasterol. Furthermore, exogenous α-pinene has been reported to reduce chlorophyll content in *Elymus nutans*, possibly through disruption of membrane homeostasis [29]. This observation is consistent with the present findings and supports a role for α-pinene in the regulation of chlorophyll content.

The present study also identified oleic acid and triolein as additional metabolites associated with sclareol-induced chlorophyll reduction. In higher plants, unsaturated fatty acids generally decline during senescence, whereas triacylglycerols (TAGs) tend to accumulate [30,31,32], indicating distinct regulatory patterns. In contrast, both unsaturated fatty acids and TAGs have been reported to accumulate in response to abiotic stresses, such as salinity and drought [33,34,35]. Together with the observed induction of *AtMYB30*, a regulator of fatty acid and lipid biosynthesis, these findings suggest that sclareol promotes the accumulation of oleic acid and triolein, at least in part through transcriptional activation of lipid-related pathways.

Furthermore, sclareol treatment increased both the accumulation of pipecolic acid and the expression of *AtSARD4*, a key gene involved in its biosynthesis. Pipecolic acid is known to accumulate in response to both biotic and abiotic stresses, including pathogen infection, drought, salinity, and chemical treatments [36,37,38]. Therefore, exogenously applied sclareol may function as a chemical elicitor that activates stress-responsive metabolic pathways, leading to enhanced pipecolic acid production.

Recent studies indicate that phytosterols, terpenoids, lipids, and pipecolic acid function within interconnected regulatory networks that govern plant growth, stress adaptation, and disease resistance [36,37,39,40,41,42,43,44,45,46]. Although these metabolites arise from distinct biosynthetic pathways, they converge on several key physiological processes, including membrane remodeling, redox homeostasis, hormone signaling, and defense responses. In both the present and previous studies, sclareol treatment promoted the accumulation of campesterol, stigmasterol, α-pinene, oleic acid, triolein, and pipecolic acid, accompanied by the upregulation of genes associated with their biosynthesis. Collectively, these findings suggest that sclareol elicits a coordinated metabolic reprogramming involving multiple biosynthetic pathways rather than activating a single downstream signaling route.

Lipids may represent a key interface between terpenoid- and pipecolic acid–associated responses because membrane composition affects signal perception, signal transduction, and cellular adaptation to stress [42,44,45]. In addition, phytosterols play important roles in membrane organization and defense-associated signaling [40,41,42,43], while terpenoids participate in multiple stress-responsive signaling pathways [20,21,39]. Therefore, the coordinated accumulation of phytosterols, terpenoids, lipids, and pipecolic acid in response to sclareol treatment supports the notion that sclareol activates an integrated metabolic network that contributes to stress adaptation and defense regulation.

Collectively, the present findings indicate that sclareol promotes coordinated metabolic and transcriptional reprogramming across multiple biosynthetic pathways, including terpenoid, lipid, and amino acid metabolism. The concurrent accumulation of phytosterols, terpenoids, lipids, and pipecolic acid may contribute to the chlorophyll reduction observed in sclareol-treated plants, pointing to a complex regulatory network that integrates metabolic and physiological responses in *Arabidopsis*.

## 4. Conclusions

**Figure 8:**
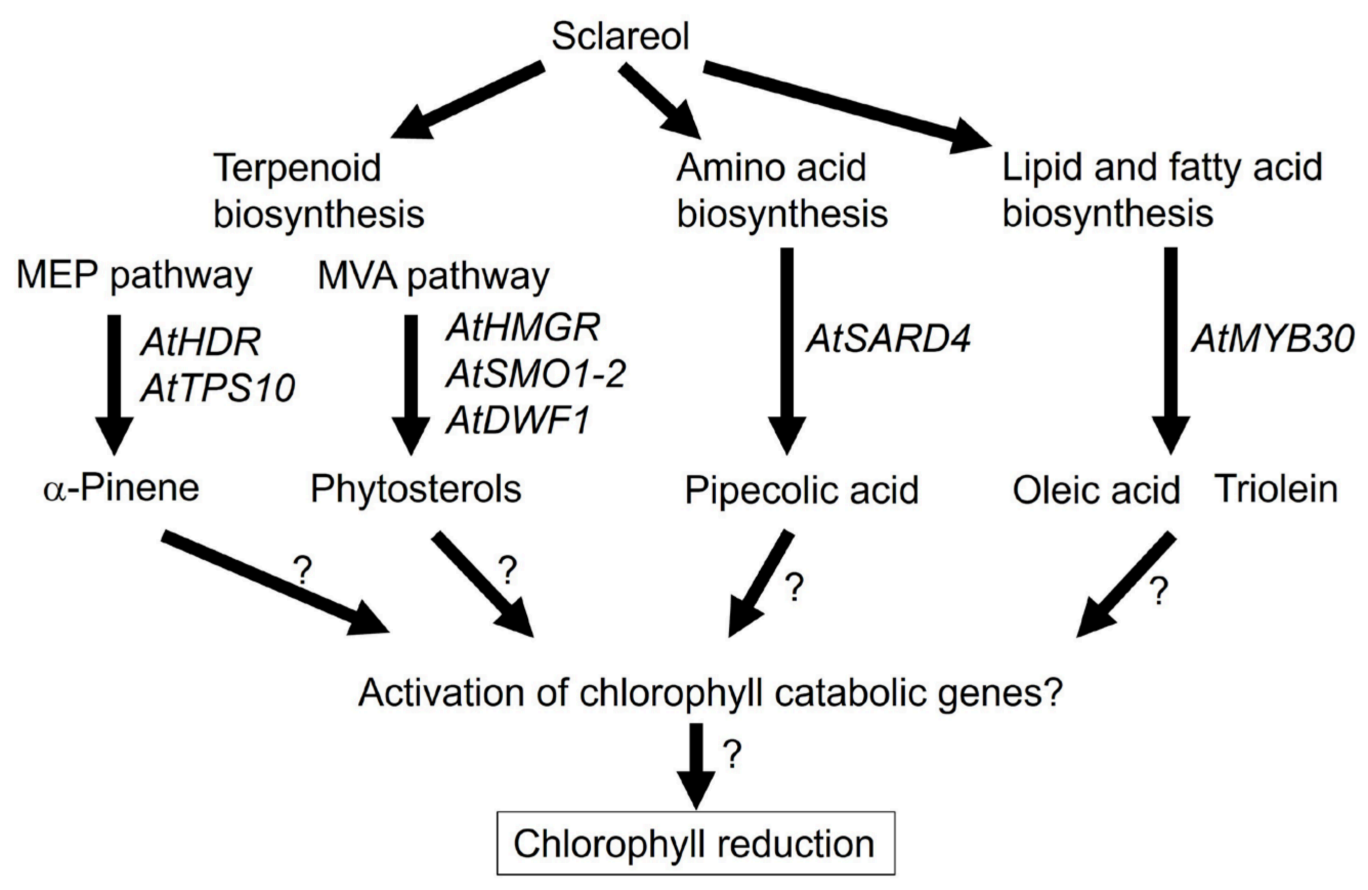
Proposed pathway for metabolite accumulation following exogenous sclareol treatment and its association with chlorophyll reduction in *Arabidopsis*. The proposed model summarizes the metabolic responses induced by sclareol treatment that lead to the accumulation of chlorophyll-reducing metabolites. The involvement of HDR, TPS10, MYB30, and SARD4 is based on the findings of the present study, whereas the involvement of HMGR, SMO1, and DWF1 is based on our previous report [19]. Abbreviations: HDR, 4-hydroxy-3-methylbut-2-enyl diphosphate reductase; TPS10, terpene synthase 10; HMGR, 3-hydroxy-3-methylglutaryl-CoA reductase; SMO1, sterol 4α-methyl oxidase 1; DWF1, sterol C-24 reductase; SARD4, SAR-DEFICIENT 4; MYB30, MYB domain protein 30.

The present study demonstrated that exogenous sclareol induces the accumulation of multiple metabolites in *Arabidopsis*, including campesterol, stigmasterol, α-pinene, oleic acid, triolein, and pipecolic acid, which are associated with chlorophyll reduction (Figure 8). These metabolites have also been implicated in plant growth and development, abiotic stress responses, and disease resistance [39,40,41,42,43,44,45,46,47]. Together with previous reports showing that sclareol inhibits plant growth, activates defense-related gene expression, and enhances disease resistance [8,14,15,16,17,18], our findings suggest that these metabolites participate in multiple physiological processes elicited by sclareol. The results further support the hypothesis that sclareol functions as a chemical elicitor capable of activating diverse metabolic pathways involved in plant stress responses.

Although the molecular mechanisms linking metabolite accumulation to chlorophyll reduction remain unclear, one possible explanation is that these metabolites promote the expression of chlorophyll catabolic genes (Figure 8), which play central roles in chlorophyll degradation [48]. This hypothesis is consistent with previous studies showing that phytohormones such as jasmonic acid and abscisic acid stimulate chlorophyll degradation through transcriptional activation of chlorophyll catabolic genes [48,49].

Further studies are needed to elucidate the signaling pathways and molecular mechanisms that connect sclareol-induced metabolite accumulation with chlorophyll degradation and other physiological responses in plants.

## 5. Materials and Methods

### 5.1. Plant Materials

*Arabidopsis thaliana* Columbia (Col-0) was used in this study and grown in a hydroponic culture system. Seeds were sown on Murashige and Skoog (MS) medium [50] supplemented with 2% (*w*/*v*) sucrose and 0.8% (*w*/*v*) agar. Plants were cultivated under a photoperiod of 8 h light/16 h dark at 22 ± 1 °C, with a photon flux density of 120 μmol m^−2^ s^−1^. Approximately 10-day-old seedlings were carefully removed from the agar medium and transferred onto a polyethylene raft containing 1-cm-diameter holes. The raft was floated on a plastic container filled with a 1000-fold diluted liquid fertilizer (Hyponex, Hyponex Japan Corp., Osaka, Japan), allowing the roots to be submerged in the nutrient solution. Plants were grown under the same environmental conditions as described above, and the nutrient solution was replaced weekly.

Unless otherwise stated, four-week-old plants were used for all experiments.

### 5.2. Chemicals

Sclareol was obtained from Sigma-Aldrich (St. Louis, MO, USA). α-Pinene, triolein, oleic acid, and pipecolic acid were purchased from Tokyo Chemical Industry Co., Ltd. (Tokyo, Japan). Sclareol, α-pinene, and oleic acid were dissolved in methanol to prepare 100 mM stock solutions, which were subsequently diluted with water to the desired concentrations. Triolein was dissolved in methanol containing 50% (*v*/*v*) dimethyl sulfoxide (DMSO) to prepare a 100 mM stock solution and diluted with water supplemented with 0.1% (*v*/*v*) DMSO and 0.1% (*v*/*v*) methanol to the required concentrations. Pipecolic acid was dissolved directly in water and diluted to the desired concentrations.

All chemical solutions were freshly prepared immediately prior to use.

### 5.3. Chemical Treatments

Hydroponically grown plants were carefully removed from the nutrient solution and transferred to 9-cm-diameter glass Petri dishes containing 40 mL of distilled water. To minimize potential effects of mechanical stress during handling, plants were preincubated for 48 h. Following preincubation, the distilled water was gently removed using a pipette, and 40 mL of treatment solution containing the indicated concentrations of each chemical was added to each dish. Plants were then incubated for an additional 48 h under the same growth conditions. After treatment, plants were used for the purification and quantification of chlorophyll content-reducing substances, chlorophyll content measurements, and total RNA extraction.

### 5.4. Purification of Chlorophyll Content-Reducing Substances

#### 5.4.1. Fractionation and Purification

Hydroponically grown *Arabidopsis thaliana* plants (approximately 500 g fresh weight) were treated by immersing their roots in either 100 µM sclareol (sclareol treatment) or 0.1% (*v*/*v*) methanol (control) for 48 h. Following treatment, whole plant tissues were extracted with five volumes of 80% (*v*/*v*) acetone at 4 °C. Hexane- and ethyl acetate-soluble fractions were subsequently prepared from the acetone extract as described previously [19].

The hexane-soluble fraction was subjected to silica gel column chromatography (53 mm i.d. × 550 mm; Wakogel C-200, FUJIFILM Wako Pure Chemical, Osaka, Japan) using a hexane-ethyl acetate solvent system with a stepwise gradient of increasing ethyl acetate concentration under gravity flow conditions. Fractions eluted with 40–50% (*v*/*v*) ethyl acetate were collected, combined, and evaporated to dryness under reduced pressure. The residue was dissolved in 80% (*v*/*v*) methanol and further purified by reversed-phase high-performance liquid chromatography (HPLC) using a C18 column (Symmetry C18, 5 µm, 4.6 × 250 mm; Waters, Milford, MA, USA). The mobile phase consisted of a methanol–water gradient (0–10 min, 0–5% methanol; 10–45 min, 5–100% methanol; 45–55 min, 100% methanol) at a flow rate of 1.0 mL min^−1^, with detection at 254 nm. Peaks with retention times of 10.2, 33.1, and 35.3 min were collected separately and evaporated to yield compounds 1, 2, and 3, respectively.

The ethyl acetate-soluble fraction was processed in a similar manner. Briefly, the fraction was separated by silica gel column chromatography (53 mm i.d. × 550 mm; Wakogel C-200) using a chloroform–methanol solvent system with a stepwise gradient of increasing methanol concentration (0–100%). The fraction eluted with 100% methanol was collected and evaporated to dryness. The residue was dissolved in 80% (*v*/*v*) methanol and subjected to reversed-phase HPLC under the conditions as described above. A peak with a retention time of 19.5 min was collected and evaporated to yield compound 4.

All fractions were evaporated to dryness, redissolved in methanol, diluted with water to the desired concentrations, and used for assays of chlorophyll content-reducing activity.

#### 5.4.2. High-Performance Liquid Chromatography (HPLC)

High-performance liquid chromatography (HPLC) was performed using a Waters 600 Delta system (Waters, Milford, MA, USA) equipped with a multi-solvent delivery pump (Model 600), a UV/Vis detector (Model 2489, Waters), Empower 2 software for data acquisition and analysis (Waters), and a vacuum degasser (PG-12 GASTORR; FLOM, Tokyo, Japan). This system was employed for the purification of active compounds.

### 5.5. Measurement of Chlorophyll Content-Reducing Activity

Six hydroponically grown *Arabidopsis thaliana* plants were placed in 9-cm-diameter glass Petri dishes containing distilled water and preincubated for 48 h. Following preincubation, the solution was carefully removed, and 40 mL of treatment solution containing the indicated concentrations of each fraction or chemical compound was added to each dish. Plants were then incubated for 48 h under the same growth conditions.

After treatment, chlorophyll was extracted from the leaves and quantified to assess chlorophyll content-reducing activity. A pool of six plants was defined as one biological replicate.

### 5.6. Chlorophyll Measurement

Chlorophyll extraction and quantification were performed as previously described [19]. Briefly, aerial tissues of *Arabidopsis* were harvested and homogenized in four volumes of 80% (*v*/*v*) acetone using a mortar and pestle on ice. The homogenate was centrifuged, and the supernatant was collected. The residual pellet was re-extracted with 80% acetone on ice, and the resulting supernatants were combined. Chlorophyll content was determined spectrophotometrically by measuring absorbance at 646 and 663 nm. Chlorophyll *a* and *b* concentrations were calculated according to previously established equations [51].

### 5.7. Structural Analyses of Active Substances

#### 5.7.1. NMR

1D- and 2D-NMR spectra (^1^H, ^13^C, HSQC, HMBC, COSY, and NOESY) were performed using Bruker Avance-NMR (600 and 800 MHz, Bruker Corporation, Billerica, MA, USA).

#### 5.7.2. Gas Chromatography–Mass Spectrometry (GC–MS)

Mass spectrometric analysis was performed using a gas chromatograph (7890A; Agilent Technologies, Santa Clara, CA, USA) coupled to a quadrupole mass spectrometer (5975C; Agilent Technologies) operated in scan mode over an *m/z* range of 50–500. Separation was achieved using a capillary column (HP-5MS; 30 m × 0.25 mm i.d., 0.25 μm film thickness; Agilent Technologies) with helium as the carrier gas at a flow rate of 1.0 mL min^−1^. The oven temperature was initially held at 50 °C for 1 min, increased to 300 °C at a rate of 2 °C min^−1^, and maintained at 300 °C for 5 min. The injector and transfer line temperatures were set at 250 °C and 280 °C, respectively. Ionization was performed by electron ionization (EI, positive mode) at 70 eV. Compounds were identified by comparing their retention times and mass spectra with those of authentic standards.

#### 5.7.3. α-Pinene (Compound 1)

Oily; ^1^H-NMR (CDCl_3_, 800 MHz): 5.18(m, 1H, H3), 2.33 (m, 1H, H7), 2.22 (m, 1H, H4), 2.16 (m, 1H, H4), 2.07 (m, 1H, H5), 1.93 (td, *J* = 1.4; 5.7, 1H, H1), 1.65 (q, *J* = 2.1, 3H, H10), 1.26 (s, 3H, H8), 1.15 (d, *J* = 8.8, 1H, H7), 0.84 (s, 3H, H9) and ^13^C-NMR data (CDCl_3_, 800 MHz): 144.5, 116.0, 47.1, 40.74, 37.98, 31.5, 31.3, 26.4, 23.0, 20.8, as described in the literature [52]; GC–MS *m/z* 136 [M]^+^ (C_10_H_16_).

#### 5.7.4. Oleic Acid (Compound 2)

Oily; 1H-NMR (CDCl_3_, 800 MHz): 5.34 (m, 2H), 2.34 (t, *J* = 7.5; 7.5, 2H), 2.01 (m, 4H), 1.63 (q, *J* = 8.0; 7.2, 2H), 1.36–1.27 (m, 20H), 0.87 (t, *J* = 7.2; 7.1, 3H) and ^13^C-NMR data (CDCl_3_, 800 MHz): 179.4, 130.0, 129.7, 33.9, 31.9, 29.7–29.0, 27.2, 27.1, 24.7, 22.7, 14.1, as described in the literature [53]; GC–MS *m/z* 282 [M]^+^ (C_18_H_34_O_2_).

#### 5.7.5. Triolein (Compound 3)

Oily; ^1^H-NMR (CDCl_3_, 800 MHz): 5.35 (m, 2H), 5.26 (m, 1H), 4.29 (q, *J* = 4.0; 11.9, 2H), 4.14 (q, *J* = 6.4; 11.9, 2H), 2.31 (m, 2H), 2.01 (m, 4H), 1.60 (m, 2H), 1.36–1.20 (m, 20H), 0.88 (t, *J* = 6.5; 7.5, 3H) and ^13^C-NMR data (CDCl_3_, 800 MHz): 130.2–127.9, 68.9, 62.1, 34.2–34.0, 29.7–29.1, 27.2, 27.1, 24.9–24.8, 14.1, as described in the literature [54]; GC–MS *m/z* 885 [M]^+^ (C_57_H_104_O_6_).

#### 5.7.6. Pipecolic Acid (Compound 4)

White powder; ^1^H-NMR (CDCl_3_, 800 MHz): 3.40 (dd, *J* = 4.0; 11.5, 1H), 3.28 (m, 1H), 2.93 (td, *J* = 3.2; 12.5, 1H), 1.86 (m, 1H), 1.81 (m, 1H), 1.64 (m, 2H), 1.57 (m, 1H) and ^13^C-NMR data (CDCl_3_, 800 MHz): 173.9, 60.6, 44.7, 28.1, 23.5, 23.2, as compared with a standard; GC–MS *m/z* 129 [M]^+^ (C_6_H_11_NO_2_).

### 5.8. Quantification of Chlorophyll Content-Reducing Substances

#### 5.8.1. α-Pinene

Quantification of α-pinene was performed with modifications to a previously reported method [55]. Briefly, 30 *Arabidopsis* plants (approximately 15 g fresh weight) were harvested and ground in liquid nitrogen using a mortar and pestle. The homogenate was extracted with five volumes of acetone at 4 °C. Ethyl decanoate was added at a known concentration as an internal standard at 4 °C. The extract was filtered through filter paper, and acetone was removed by evaporation. The resulting aqueous phase was extracted three times with ethyl acetate. The combined organic phases were dried over anhydrous sodium sulfate and evaporated to dryness. The residue was dissolved in ethyl acetate, and aliquots were subjected to gas chromatography–mass spectrometry (GC–MS) analysis.

GC–MS was performed using a gas chromatograph (7890A; Agilent Technologies, Santa Clara, CA, USA) coupled to a quadrupole mass spectrometer (5975C; Agilent Technologies) operated in selected ion monitoring (SIM) mode using *m/z* values of 136 and 93. Separation was achieved on a capillary column (HP-5MS; 30 m × 0.25 mm i.d., 0.25 μm film thickness; Agilent Technologies) with helium as the carrier gas at a flow rate of 1.0 mL min^−1^. The oven temperature was held at 40 °C for 2 min, increased to 80 °C at 2 °C min^−1^, ramped to 300 °C at 25 °C min^−1^, and maintained at 300 °C for 5 min. The injector and transfer line temperatures were set at 250 °C and 280 °C, respectively. Electron ionization (EI, positive mode) was applied at 70 eV.

A pool of 30 plants was defined as one biological replicate. Recovery of the internal standard ranged from 45 to 55%, and all quantified values were corrected for losses.

#### 5.8.2. Pipecolic Acid

Quantification of pipecolic acid was performed with modifications to a previously described method [56]. Briefly, three *Arabidopsis* plants (approximately 1.5 g fresh weight) were harvested, flash-frozen in liquid nitrogen, and homogenized using a bead mill homogenizer (MS-100R; TOMY Corp., Tokyo, Japan) at 4000 rpm for 90 s at 4 °C in polypropylene tubes containing two stainless steel beads (5 mm diameter). Five volumes of 80% (*v*/*v*) methanol containing norvaline at a known concentration as an internal standard were added to the homogenate, and the mixture was incubated on ice for 1 h. After centrifugation at 10,000× *g* for 15 min at 4 °C, the supernatant was collected and diluted with a buffer consisting of 0.5 M ammonium formate (pH 4.0):acetonitrile:water:methanol (10:60:10:20, *v*/*v*/*v*/*v*). Aliquots were subjected to ultra-performance liquid chromatography–tandem mass spectrometry (UPLC–MS/MS) analysis.

UPLC–MS/MS analyses were performed using an ACQUITY H-Class system (Waters, Milford, MA, USA) coupled to a tandem quadrupole mass spectrometer (TQ-S micro; Waters). Separation was achieved on a reversed-phase column (Unison UK-C18 HT; 2.0 mm i.d. × 100 mm, 3 μm; Imtakt, Kyoto, Japan) using the same mobile phase at a flow rate of 0.4 mL min^−1^, with the column temperature maintained at 35 °C. Mass spectrometric detection was conducted under the following conditions: electrospray ionization in positive mode (ESI+), capillary voltage 3.5 kV, cone gas flow 50 L h^−1^, desolvation gas (N_2_) flow 1000 L h^−1^, desolvation temperature 350 °C, cone voltage 25 V, and collision energy 20 eV. Quantification was performed in multiple reaction monitoring (MRM) mode by monitoring the transition from *m/z* 130 (precursor ion) to *m/z* 84 (product ion).

A pool of three to five plants was defined as one biological replicate.

#### 5.8.3. Oleic Acid and Triolein

Quantification of oleic acid and triolein was performed with modifications to previously reported methods [47,57]. Briefly, 20 *Arabidopsis* plants (approximately 10 g fresh weight) were harvested and ground in liquid nitrogen using a mortar and pestle. Lipids were extracted by adding 10 volumes (100 mL) of chloroform–methanol (2:1, *v*/*v*) containing pentadecanoic acid and glycerol triheptadecanoate at known concentrations as internal standards. The extract was transferred to an Erlenmeyer flask, and 40 mL of ammonium acetate solution (chloroform:methanol:300 mM ammonium acetate, 30:67:3, *v*/*v*/*v*) was added. The mixture was shaken at 180 rpm for 1 h, followed by centrifugation to separate phases. The organic phase containing fatty acids and lipids was collected and evaporated to dryness. The residue was dissolved in methanol, and aliquots were diluted with buffer A (acetonitrile:10 mM ammonium acetate, 95:5, *v*/*v*) prior to analysis.

UPLC–MS/MS analysis was performed using buffer A and buffer B (acetonitrile:10 mM ammonium acetate, 50:50, *v*/*v*) as mobile phases. Separation was achieved on a hydrophilic interaction liquid chromatography (HILIC) column (CORTECS; 2.1 mm i.d. × 100 mm, 1.6 μm; Waters, Milford, MA, USA) with a gradient elution (0–6 min, 0.1–20% buffer B; 6–8 min, 20–80% buffer B; 8–12 min, 0.1% buffer B) at a flow rate of 0.4 mL min^−1^. The column temperature was maintained at 35 °C. Mass spectrometric detection was carried out using electrospray ionization in positive mode (ESI+) under the following conditions: capillary voltage 2.0 kV, cone gas flow 50 L h^−1^, desolvation gas (N_2_) flow 1000 L h^−1^, desolvation temperature 350 °C, cone voltage 25 V, and collision energy 20 eV. Quantification was performed in multiple reaction monitoring (MRM) mode by monitoring the transitions *m/z* 281.3 → 281.3 for oleic acid and *m/z* 903.0 → 603.5 for triolein.

A pool of 15 to 20 plants was defined as one biological replicate.

### 5.9. Total RNA Extraction

Total RNA was extracted from plant tissues using TRIzol reagent (Thermo Fisher Scientific, Waltham, MA, USA) according to the manufacturer’s instructions. The extracted RNA was further purified using an RNeasy Plant Mini Kit (Qiagen, Venlo, The Netherlands) following the manufacturer’s protocol. The purified RNA was subsequently used for real-time quantitative PCR (qPCR) analyses.

### 5.10. Real-Time Quantitative PCR (qPCR)

Real-time qPCR was performed as previously described [19] using gene-specific primers (Supplementary Table S1). The expression levels of target genes were normalized to *AtACTIN2*. Relative gene expression was calculated using the 2^–ΔΔCt^ method [58].

### 5.11. Statistical Analyses

Statistical analyses were performed using R software (version 4.4.2; R Development Core Team). Differences among multiple groups were evaluated by one-way analysis of variance (ANOVA) followed by Tukey’s post hoc test. Comparisons between two groups were conducted using Student’s *t*-test. Dunnett’s test was applied for the analysis of data presented in Figure 2C and Supplementary Figure S1.

## Data Availability

Data supporting the conclusions of this study are included in the manuscript.

## Supplementary Materials

The following supporting information can be downloaded at: https://www.mdpi.com/article/10.3390/plants15152301/s1, Figure S1: Flow diagram of the purification and isolation of chlorophyll content-reducing substances; Table S1: Primers used for quantitative real-time PCR analysis.

## Abbreviations

The following abbreviations are used in this manuscript:

HPLC	High-performance liquid chromatography
NMR	Nuclear magnetic resonance
UPLC	Ultraperformance liquid chromatography
MS	Mass spectrometry
MEP	Methylerythritol 4-phosphate
IPP	Isopentenyl phosphate
HDR	4-Hydroxy-3-methylbut-2-enyl diphosphate reductase
TPS	Terpenoid synthase
GDP	Geranyl diphosphate
MVA	Mevalonic acid
TAG	Triacylglycerol
DMAPP	Dimethylallyl diphosphate
