# Supplementary material for: Identification of Endogenous Substances Involved in Sclareol-Induced Chlorophyll Reductions in Arabidopsis
Source: Plants (Basel). 2026 Jul 27;15(15):2301. doi: 10.3390/plants15152301 (PMC13468004; doi:10.3390/plants15152301)
Supplement: Supplementary file 1 [file plants-15-02301-s001.zip › plants-4405542-supplementary.pdf]

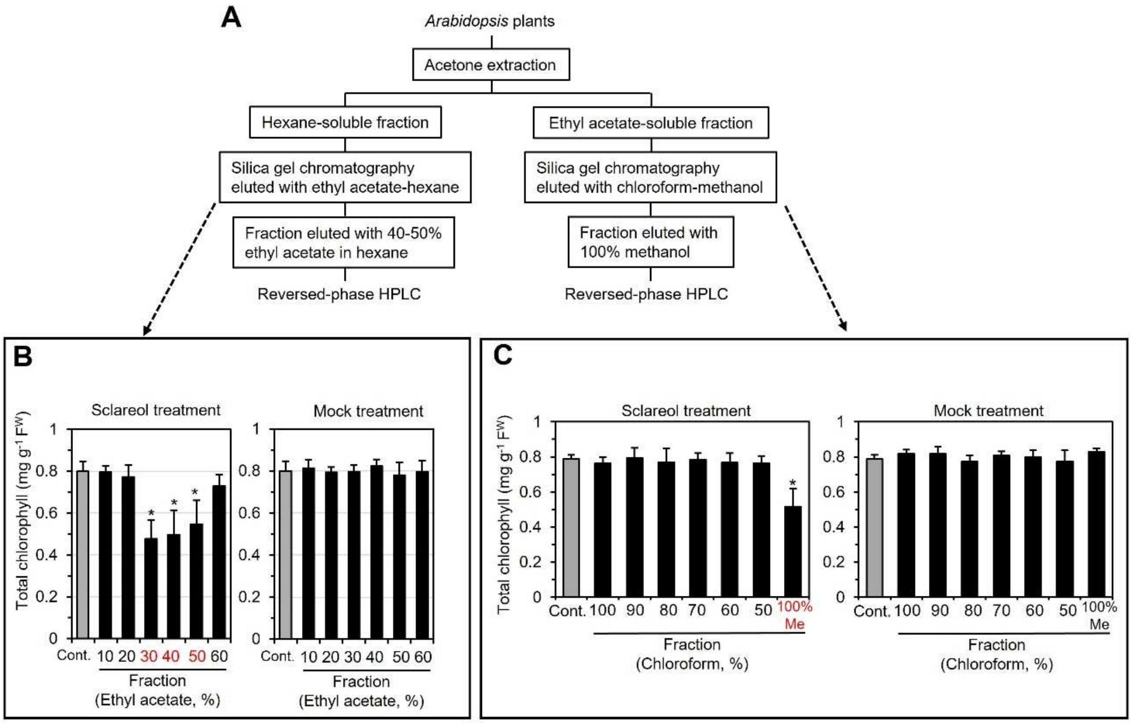

Supplementary Figure S1. Flow diagram of the purification and isolation of chlorophyll content-reducing substances. (**A**) Schematic representation of extraction, fractionation, and purification procedures. (**B**, **C**) Chlorophyll content–reducing activity of fractions obtained from the hexane-soluble (**B**) and ethyl acetate–soluble (**C**) extracts. The control treatment consisted of 0.1% (*v/v*) methanol. Data are presented as means ± standard deviation (SD) of six biological replicates. Asterisks indicate significant differences compared with the control (0.1% methanol; Dunnett's test, $P < 0.05$).

Supplementary Table S1. Primers used for quantitative real-time PCR analysis.

Gene name	AGI code	Forward	Reverse
AtPDR12	At1g15520	CTTTCGCTCAGGTTTTCATCG	CTTCACCGCCGTCCACTC
AtHDR	At4g34350	GACGCTACTCAAGAGCGACA	TGAATTCCATCCGCCAACCA
AtTPS10	At2g24210	GCTTCGACGTCCTCAGTCTT	CGCTGAACTGTGGGAGACTT
AtMYB30	At3g28910	GGAAAGGCGACTTCAAACCG	AATGATTGGTGCAACGGCAG
AtSARD4	At5g52810	GTGTTTAAGTCCGTCGGTTCG	AACGGCTGAGGTAAGTCTCG
Atactin2	At3g18780	GGTAACATTGTGCTCAGTGGTGG	GGTGCAACGACCTTAATCTTCAT
